# The Efficacy of Computerized Cognitive Behavioral Therapy for Depressive and Anxiety Symptoms in Patients With COVID-19: Randomized Controlled Trial

**DOI:** 10.2196/26883

**Published:** 2021-05-14

**Authors:** Zhifen Liu, Dan Qiao, Yifan Xu, Wentao Zhao, Yang Yang, Dan Wen, Xinrong Li, Xiaoping Nie, Yongkang Dong, Shiyou Tang, Yi Jiang, Ying Wang, Juan Zhao, Yong Xu

**Affiliations:** 1 Department of Psychiatry The First Hospital of Shanxi Medical University Taiyuan China; 2 Department of Medical Service Chongqing Public Health Medical Center Chongqing China; 3 The Fourth People's Hospital of Taiyuan Taiyuan China; 4 Department of Psychiatry Chongqing University Three Gorges Hospital Chongqing China; 5 Department of Respiratory and Critical Disease Medicine The First Hospital of Shanxi Medical University Taiyuan China; 6 Department of Geriatrics The First Hospital of Shanxi Medical University Taiyuan China

**Keywords:** mental health, depression, anxiety, COVID-19, treatment, cCBT, computerized cognitive behavioral therapy

## Abstract

**Background:**

The prevalence of depressive and anxiety symptoms in patients with COVID-19 is higher than usual. Previous studies have shown that there are drug-to-drug interactions between antiretroviral drugs and antidepressants. Therefore, an effective and safe treatment method was needed. Cognitive behavioral therapy (CBT) is the first-line psychological therapy in clinical treatment. Computerized CBT (cCBT) was proven to be an effective alternative to CBT and does not require face-to-face therapy between a therapist and the patient, which suited the COVID-19 pandemic response.

**Objective:**

This study aims to evaluate the efficacy of the cCBT program we developed in improving depressive and anxiety symptoms among patients with COVID-19.

**Methods:**

We customized a cCBT program focused on improving depressive and anxiety symptoms among patients with COVID-19, and then, we assessed its effectiveness. Screening was based on symptoms of depression or anxiety for patients who scored ≥7 on the Hamilton Depression Rating Scale (HAMD_17_) or the Hamilton Anxiety Scale (HAMA). A total of 252 patients with COVID-19 at five sites were randomized into two groups: cCBT + treatment as usual (TAU; n=126) and TAU without cCBT (n=126). The cCBT + TAU group received the cCBT intervention program for 1 week. The primary efficacy measures were the HAMD_17_ and HAMA scores. The secondary outcome measures were the Self-Rating Depression Scale (SDS), Self-Rating Anxiety Scale (SAS), and Athens Insomnia Scale (AIS). Assessments were carried out pre- and postintervention. The patients’ symptoms of anxiety and depression in one of the centers were assessed again within 1 month after the postintervention assessment.

**Results:**

The cCBT + TAU group displayed a significantly decreased score on the HAMD_17_, HAMA, SDS, SAS, and AIS after the intervention compared to the TAU group (all *P*<.001). A mixed-effects repeated measures model revealed significant improvement in symptoms of depression (HAMD_17_ and SDS scores, both *P*<.001), anxiety (HAMA and SAS scores, both *P*<.001), and insomnia (AIS score, *P*=.002) during the postintervention and follow-up periods in the cCBT + TAU group. Additionally, the improvement of insomnia among females (*P*=.14) and those with middle school education (*P*=.48) in the cCBT + TAU group showed no significant differences when compared to the TAU group.

**Conclusions:**

The findings of this study suggest that the cCBT program we developed was an effective nonpharmacological treatment for symptoms of anxiety, depression, and insomnia among patients with COVID-19. Further research is warranted to investigate the long-term effects of cCBT for symptoms of anxiety, depression, and insomnia in patients with COVID-19.

**Trial Registration:**

Chinese Clinical Trial Registry ChiCTR2000030084; http://www.chictr.org.cn/showprojen.aspx?proj=49952

## Introduction

In the past decades, the effects of physical and psychological distress have increased with each successive public health emergency, such as with the severe acute respiratory syndrome (SARS) in 2003 [1], the Middle East Respiratory Syndrome (MERS) [2] in 2012, the Ebola virus disease in 2014 [3], and COVID-19 in 2020 [4]. COVID-19, caused by a novel coronavirus (SARS-CoV-2) [5], continues to spread worldwide. On January 30, 2020, the World Health Organization Emergency Committee declared a global health emergency based on growing case notification rates at Chinese and international locations [6]. At present, there have been nearly 30 million confirmed cases and nearly 1 million deaths worldwide [7].

The COVID-19 epidemic is similar to SARS and MERS [8] but has spread more quickly and efficiently [9]. In the acute stage of SARS and MERS, the prevalence of psychological distress among confirmed patients was 63.0%; common symptoms included insomnia (41.9%), anxiety (35.7%), and depression (32.6%) [10]. A cross-sectional study on psychological distress in patients with COVID-19 showed that the prevalence of symptoms of depression and anxiety was 35.9% and 38.5%, respectively [11].

The common clinical symptoms of patients with COVID-19 include cough, fever, fatigue, hypoxia, and occasionally gastrointestinal infection [12]. Moreover, the patients experienced various stressors during isolation, including but not limited to drug side effects, a fear of severe disease consequences, a fear of infecting others, and an inability to get correct information in a timely manner [13]—all of which may lead to several psychological distresses including anxiety, depression, and insomnia [8,14]. A lack of timely intervention in psychological distresses may affect the quality of life for patients in the future. As a survey of SARS survivors showed, 42.5% were diagnosed with at least one psychiatric disorder 4 years following the outbreak, with the most common diagnoses being posttraumatic stress disorder (PTSD; 54.5%) and major depressive disorder (39.0%). Further, 40.3% of survivors experienced chronic fatigue [15].

Early, brief, and trauma-focused mental health services may have the potential to reduce or delay the development of adverse outcomes [16]. An effective method may be cognitive behavioral therapy (CBT), which is widely used to treat mild to moderate depression and anxiety [17]. However, traditional CBT requires a face-to-face implementation, which is inconvenient for patients with COVID-19 who are quarantined in hospitals. Computerized CBT (cCBT), as an alternative mode of delivery, can make up for this deficiency. There is substantial evidence that cCBT is an effective alternative to CBT for the treatment of mild to moderate depression and anxiety [18-20]. Compared to CBT, cCBT offers a range of benefits. cCBT can be accessed with a computer, smartphone, or tablet without the need for face-to-face therapy, and this is especially key during a pandemic to reduce the risk of spreading infection [21]. cCBT does not require appointments, allowing instead for time flexibility and cost-effectiveness [22]. The privacy of patients is better protected, and the stigma of face-to-face therapy is mitigated [23].

Thus, to solve the psychological problems that may occur during the epidemic, we independently developed a cCBT program for patients with COVID-19. The program is a brief self-guided, online psychological intervention that consists of cognitive training, cognitive consolidation, and behavioral therapy via video and animation content, all of which is easy to understand and operate. It may comprehensively regulate mental distress that includes anxiety, depression, panic, and fear among patients with COVID-19; it may also help patients build confidence as they confront the disease.

Moreover, evidence from epidemiological studies revealed the relationship between gender, age, education level, and severity of mental problems [24]. Females and relatively high educational level were found to be predictors of mental distress of adults 1 month in the COVID-19 epidemic [25]. Additionally, several studies also suggest differences between men and women in response mechanisms to psychological distress. Men tend to *externalize* their distress by directing action outward, while women tend to *internalize* their distress by directing action inward [26,27]. Such gender difference may influence the treatment response [28]. Other studies have shown that the patients’ age and educational level at the beginning of treatment may affect the final outcomes [29].

Based on all these factors, we conducted a multicenter, randomized, placebo-controlled clinical trial to evaluate the efficacy of the cCBT program. The program was developed with the aims of improving the symptoms of depression and anxiety among patients with COVID-19. We hypothesized greater symptom improvement with respect to mitigating anxiety and depression in the intervention group compared to the treatment as usual (TAU) group at both the postintervention and follow-up periods. We also hypothesized that online self-help may not be suitable for all patients, and the overall responses in mental problems may vary by gender, age, and educational level.

## Methods

### Trial Design

This was a prospective, multicenter, two-arm (allocation ratio was 1:1), parallel group; further, it was a nonblinded randomized controlled trial that was registered at the Chinese Clinical Trial Registry with the study number ChiCTR2000030084. Participants were recruited from five hospitals in China from March 2020 to June 2020. Eligible participants were screened for symptoms of anxiety or depression. Patients with symptoms of anxiety, depression, or both were included because these two symptoms often occur simultaneously. Patients were then randomly allocated to a 1-week course of the cCBT program we developed in addition to TAU (cCBT + TAU group), or they were randomly allocated to the control condition, the TAU group, when they completed the baseline assessment. The outcomes of both groups were measured twice—at the baseline and post intervention. To investigate the relative continuous effects of cCBT on symptoms of depression and anxiety, the outcomes of both groups in one of the sites were measured again 1 month after the postintervention.

### Participants and Recruitment

The participants were recruited from the following five hospitals: Affiliated Tongji Hospital of Huazhong University of Science and Technology, Wuhan Institute for Tuberculosis Control, Central Hospital of Chongqing Three Gorges, Chongqing Public Health Medical Center, and the Fourth People’s Hospital of Taiyuan. The inclusion criteria those who were aged between 18-75 years (including threshold), regardless of gender; were under isolation observation while diagnosed with a mild or common type of COVID-19 and could cooperate to complete a corresponding psychological intervention; had mild to moderate depressive or anxiety symptoms as defined by the 17-item Hamilton Depression Rating Scale (HAMD_17_) score≥7 or the Hamilton Anxiety Scale (HAMA) score≥7; had sufficient compliance to complete the experiment according to the protocol; and informed consent was provided by patients and (if necessary) guardians. The exclusion criteria were patients who were clearly diagnosed with a psychiatric disorder, including depression, bipolar disorder, etc, in the 6 months prior to their diagnosis of COVID-19; patients with psychotic symptoms; patients with HAMD_17_ score≥24 or HAMA score≥21; patients with a high risk of suicide, defined as having a history of attempts by suicide in the 6 months prior to the study or who scored more than 3 on item three (suicide item) of the HAMD_17_ scale; patients with organic mental disorders; patients with substance abuse or dependence; patients undergoing treatment currently (pharmacological or psychological) for mental health problems; or patients presenting other conditions that the researchers believed were not suitable for this clinical trial.

The researcher explained to the eligible patients they had been introduced to the study because they were experiencing mental symptoms related to the burden of COVID-19 and that the intelligent therapy via the cCBT program we developed may help to reduce these distresses. Patients were also told that the aim of the study was to find out whether receiving this intelligent therapy might help them to relieve these mental symptoms linked to COVID-19. Participants were informed that they had a 50% chance of receiving the cCBT intervention and that the study would be offered as an additional source of support for free, not as a replacement or restriction to their current physical care. Besides, they would be provided with free medical consultation for a period of 3 years after discharge.

### Randomization

Resman, a public management platform for clinical trial management of China’s clinical trial registration agency, was used to carry out random grouping; the allocated sequence was kept by the administrator. Patients with mild to moderate anxiety or depression were randomized to the cCBT + TAU group or to the TAU group at a 1:1 ratio. Randomization was balanced using a block size of four and was stratified by institution.

### Intervention

#### The cCBT Program

The program is a remote intervention model based on cCBT that we developed. The system can systematically intervene in patients’ cognition, emotions, and behavior through an offline mobile terminal. It is easy to understand and operate, so patients can complete the work with the help of nonpsychological professionals or with self-guidance. Participants in the cCBT + TAU group were informed of how to access the program and use it during the study after registration.

Compared to other forms of digital CBT for anxiety and depression, the most unique feature of the cCBT program we developed is its targeting of patients with COVID-19. Components of the intervention were presented to patients with COVID-19 with anxiety and depressive symptoms through computer-based, visually attractive, and interactive examples, exercises, and videos. Given that the focus of CBT is on patients’ irrational cognition. The first module of cCBT, called the cognitive therapy module, aims to minimize or even eliminate patients’ negative thoughts about COVID-19. This part is presented in videos to participants. It mainly includes rational cognition of COVID-19, stress status during isolation, and psychosomatic mechanisms under stress in addition to sleeping management and education about status post isolation.

To further deepen the correct cognition of patients, we set up a cognitive consolidation module. Unlike the first module, this part is presented in the form of a miniature game, which undoubtedly increases the flexibility and interest of the intervention. The participants are required to answer questions based on videos related to the cognitive therapy module.

For the last part, the behavioral therapy module is targeted at teaching methods of regulating negative emotions. This module is also presented in the video to guide participants in relaxation training. Information about the following three relaxation training methods was provided:

1. Relaxation mental imagery training: The patients are taught to subjectively follow their own ideas and imagine some pleasant situations such as prairies, streams, trees, and greenery of native mountains. During the whole training process, patients are completely in a relaxed state with gentle and even breathes. With the vivid image gradually becoming clear in their mind, the patients will feel a warm current running through the whole body. It is during these moments that patients can now enter a deep state of relaxation benefiting both body and mind.
2. Mindfulness meditation: The goal of this training is to improve the ability of self-regulation. Using the training of concentration and relaxation, it requires the patients to consciously maintain attention on the current internal or external experience without making any judgments about it.
3. Counting meditation: Patients with COVID-19 are generally unable to relax due to the fear of the highly contagious disease. Based on this, the training guides the patients to focus on the breath while meditating. The patient’s physical and mental state will gradually calm down, and the ability to focus will continue to improve during this period of training.

This experimental treatment was delivered through more than 10 minutes of self-directed individual therapy per day for 1 week at each trial center. The program is installed on an iPad and is only available to research therapists. After the therapist shows the patients how to use the system, the patients can begin their journey of *self-help intervention*.

#### Treatment as Usual

TAU consisted of periodic psychological assessments, general psychological support, and consultations discussing overall well-being and disease activity. Patients whose assessment results suggested a certain risk to themselves or others at the time of each assessment were to be treated by a professional psychiatrist and were withdrawn from this study.

### Measures

#### Data Collection Procedure

Demographic and clinical characteristics including age, gender, and education levels (years) were collected at baseline. HAMD_17_ and HAMA scores were determined by trained researchers at the baseline, postintervention, and 1-month follow-up (hosted by only one of the centers). Secondary outcome measures were also recorded at the same point. These measures included the Self-Rating Depression Scale (SDS), the Self-Rating Anxiety Scale (SAS), and the Athens Insomnia Scale (AIS).

#### Outcome

Before the study, a questionnaire was completed by the participants including demographic and clinical characteristics. The primary efficacy outcomes were HAMD_17_ and HAMA, both well-established interviewer-rated measures of depression and anxiety severity, respectively, from baseline to the end of treatment, and to the 1-month follow-up. For HAMD_17_ [30], items are scored on a 5-point Likert scale (0-4) for a total score range from 0 to 68, with higher scores indicating more depressive symptoms. In the study sample, the total Cronbach alpha coefficient of HAMD17 was .89 (95% CI 0.86-0.92). Similarly, HAMA [31] consists of 14 items on a 5-point Likert scale (0-4) for a total score range from 0 to 56, with higher scores indicating more anxiety symptoms. Additionally, the internal consistency of this questionnaire was also good (α=.87, 95% CI 0.82-0.90). Given the severity of the COVID-19 epidemic, all assessors for HAMD_17_ and HAMA scales from different centers were trained by one professional psychological assessment specialist through online conferences. The interviewer reliability is both high for HAMD_17_ (intraclass correlation coefficient [ICC]=0.91, 95% CI 0.78-0.98) and HAMA (ICC=0.90, 95% CI 0.75-0.98).

Secondary end points for evaluating the efficacy of cCBT were SDS to assess self-rated depressive symptoms, SAS to assess anxiety symptoms, and AIS to assess insomnia symptoms. SDS [32] contains 20 items that reflect subjective feelings of depression. It is rated on a 4-point Likert scale (from 1, “no or a little of the time,” to 4, “most of the time or all the time”), containing 10 symptom positive items and 10 symptom negative items. SAS [33] is also composed of 20 items and is rated on a 4-point Likert scale (from 1, “no or a little of the time,” to 4, “most of the time or all the time”). Higher scores reflect more severe anxiety symptoms. The last secondary outcome measure is AIS [34], a validated brief questionnaire for a total score range from 0 to 24. Each item is measured on a 4-point Likert scale, with total scores between 4 and 6 representing suspicious symptoms of insomnia, scores higher than 6 representing insomnia, and scores less than 4 representing no insomnia. All three scales have been shown to have high internal consistency in this study sample (SDS: α=.93, 95% CI 0.90-0.96; SAS: α=.92, 95% CI 0.88-0.95; AIS: α=.87, 95% CI 0.81-0.92).

### Sample Size

To estimate the sample size, a pilot study was conducted for measuring the HAMD_17_ and HAMA score in 30 patients who received TAU. The standard deviation (σ) of the HAMD_17_ and HAMA score in this group were both 10.50. Additionally, based on the previous studies on such interventions for depressive or anxiety symptoms [35-38], we expected to achieve a value δ≥5 in the level of depressive and anxiety symptoms with the cCBT intervention. Assuming a 5% significance level, two-tailed, and a power of 90%, a sample size of 188 patients (ie, 94 per group) was estimated. To include an estimated dropout rate of 25%, the entire sample size was increased from 188 to 250 participants (ie, 125 per group).

### Statistical Analysis

SPSS software version 22.0 (IBM Corp) was used for all statistical analyses. Descriptive statistics were conducted for demographic and clinical characteristics in each treatment group. The measurement data were described by means and SDs. Two-sample *t* tests and chi-square or Fisher exact tests were used where appropriate, and these assessed demographic variable differences between the cCBT + TAU group and the TAU group. To confirm the improvements in each symptom at postintervention, the baseline and postintervention results of the dependent variables were analyzed using the paired *t* test, whereas the two-sample *t* test was used to detect differences between the treatment and control groups. Moreover, to explore the influence of gender, age, and education level on treatment response, we conducted a subgroup analysis to examine the changes in the scores of patients with different gender, age, and education level. For patients who finished the follow-up assessment, a mixed-effects model for repeated measures (MMRM) was used to compare data obtained at the baseline, postintervention, and follow-up periods between the cCBT + TAU group and the TAU group. Post hoc analysis was performed using the Bonferroni multiple comparison test. All tests were two-sided, and a *P* value less than .05 was considered statistically significant.

## Results

**Figure 1:**
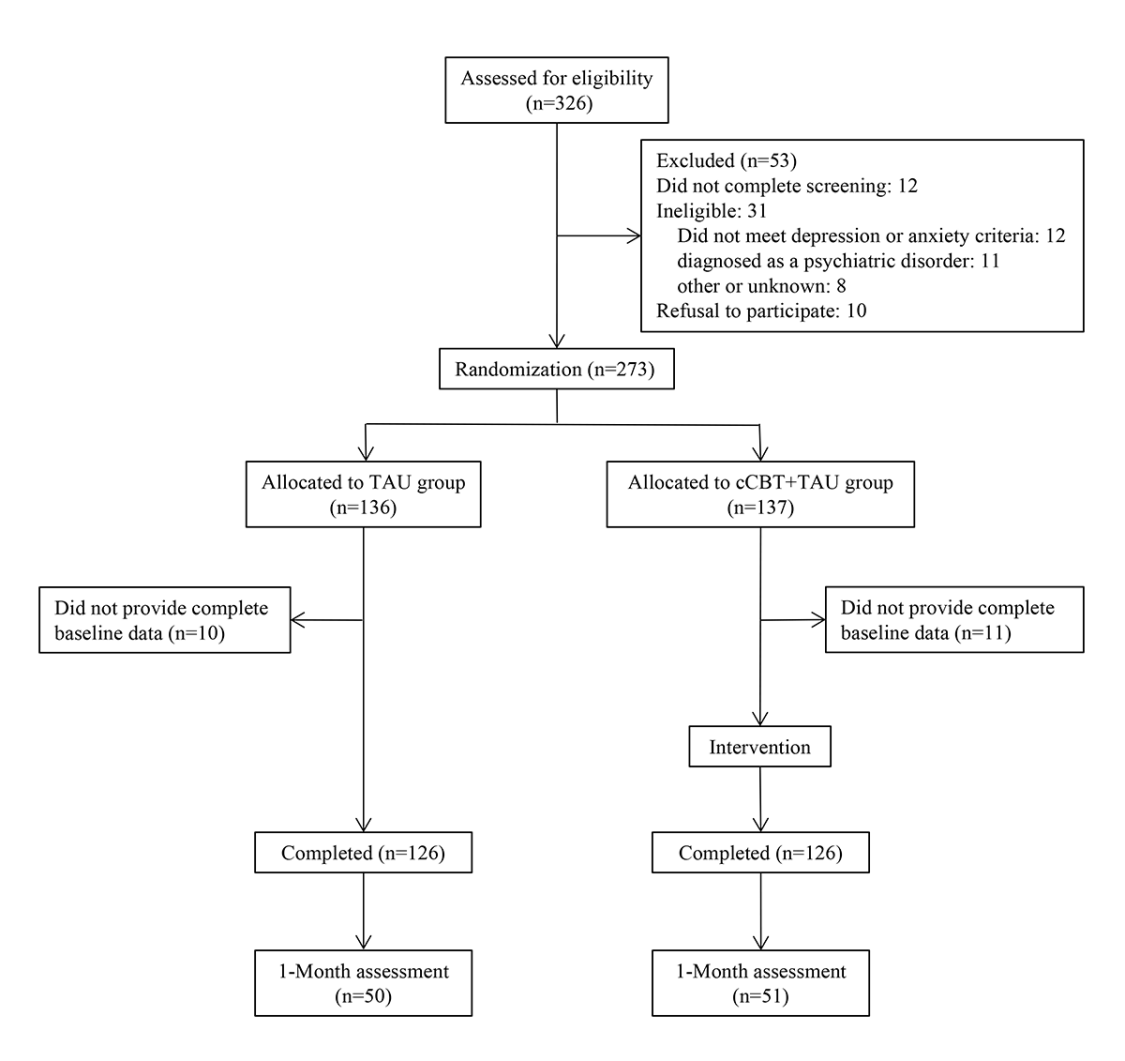
Flow diagram of the study. cCBT: computerized cognitive behavioral therapy; TAU: treatment as usual.

A total of 326 patients with COVID-19 from five centers agreed to participate to the program. There were 273 participants that completed screening (see Figure 1 for details on reasons for exclusion); of these, 21 did not provide the complete baseline data and were therefore excluded. Thus, the analyzable sample consisted of the remaining 252 participants, who were randomly assigned to either the cCBT + TAU group (n=126) or the TAU group (n=126). All participants completed the assigned intervention (Figure 1). As shown in Table 1, no significant differences were found in the demographic characteristics between the cCBT + TAU group and the TAU group. None of the participants were taking any psychiatric medication at baseline and during the intervention. For physical symptoms of COVID-19, all patients were given corresponding treatments according to the Diagnosis and Treatment Protocol for COVID-19 issued by the National Health Commission, mainly including antiviral therapy, antibacterial therapy, and traditional Chinese medicine treatment. The results for the efficacy variables are presented in Table 2. Compared to the TAU group, the cCBT + TAU group showed significant improvement in both the primary outcomes of HAMD_17_ and HAMA scores and the secondary outcomes of SDS, SAS, and AIS scores (all *P*<.001). Additionally, there were significant differences between the two groups in HAMD_17_, HAMA, SDS, and SAS scores post intervention (all *P*<.001).

**Table 1:** Baseline characteristics of the full sample and follow-up sample.

Characteristics			cCBT^a^ + TAU^b^ group (n=126)	TAU group (n=126)	Difference (95% CI)	*t* test (*df*)	Chi-square (*df*)	*P* value	Cohen *d* or φ
**The full sample**									
	Patients, n		126	126	N/A^c^	N/A	N/A	N/A	N/A
	**Sex, n**				N/A	N/A	1.65 (1)	.20	0.08^d^
		Male	70	80					
		Female	56	46					
	Age (years), mean (SD)		43.76 (14.31)	41.52 (11.51)	2.24 (–0.98 to 5.46)	1.37 (250)	N/A	.17	0.17
	Education (years), mean (SD)		10.68 (3.91)	10.67 (4.39)	0.02 (–1.01 to 1.05)	0.03 (250)	N/A	.98	<0.01
	**Site, n**				N/A	N/A	N/A	N/A	N/A
		Affiliated Tongji Hospital of Huazhong University of Science and Technology	20	19					
		Wuhan Institute for Tuberculosis Control	13	9					
		Central Hospital of Chongqing Three Gorges	26	25					
		Chongqing Public Health Medical Center	55	57					
		The Fourth People's Hospital of Taiyuan	12	16					
**Follow-up sample**									
	Patients, n		51	50	N/A	N/A	N/A	N/A	N/A
	**Sex, n**				N/A	N/A	0.11 (1)	.74	0.03^d^
		Male	31	32					
		Female	20	18					
	Age (years), mean (SD)		42.26 (12.66)	42.10 (10.58)	0.16 (–4.41 to 4.74)	0.07 (99)	N/A	.94	0.01
	Education (years), mean (SD)		10.57 (4.21)	10.72 (4.79)	–0.15 (–1.92 to 1.61)	–0.17 (99)	N/A	.86	–0.03

^a^cCBT: computerized cognitive behavioral therapy.

^b^TAU: treatment as usual.

^c^N/A: not applicable.

^d^Indicates a φ value.

**Table 2:** Changes in the primary and secondary outcomes after the intervention between groups.

Measure			cCBT^a^ + TAU^b^ group (n=126)		TAU group (n=126)		Difference (95% CI)		*P* value^c^		Cohen *d*
**HAMD_17_^d^**											
	Baseline, mean (SD)	15.13 (3.33)		15.52 (3.43)		–0.39 (–1.23 to 0.45)		.36		–0.12	
	Postintervention, mean (SD)	8.19 (3.54)		15.20 (3.64)		–7.01(–7.90 to –6.12)		<.001		–1.95	
	Difference (95% CI)	6.94 (6.34 to 7.54)		0.32 (–0.04 to 0.68)		N/A^e^		N/A		N/A	
	*P* value^f^	<.001		.08		N/A		N/A		N/A	
	Cohen *d*	2.02		0.16		N/A		N/A		N/A	
**HAMA^g^**											
	Baseline, mean (SD)	14.52 (3.13)		13.97 (2.72)		0.56 (–0.17 to 1.28)		.13		0.18	
	Postintervention, mean (SD)	7.79 (3.60)		13.63 (3.24)		–5.84 (–6.69 to –4.99)		<.001		–1.70	
	Difference (95% CI)	6.73(6.13 to 7.33)		0.33 (–0.09 to 0.75)		N/A		N/A		N/A	
	*P* value	<.001		.12		N/A		N/A		N/A	
	Cohen *d*	1.97		0.14		N/A		N/A		N/A	
**SDS^h^**											
	Baseline, mean (SD)	45.89 (8.78)		45.66 (8.28)		0.23 (–1.89 to 2.35)		.83		0.03	
	Postintervention, mean (SD)	31.95 (6.88)		44.87 (7.48)		–12.92 (–14.70 to –11.14)		<.001		–1.79	
	Difference (95% CI)	13.94 (12.73 to 15.15)		0.79 (–0.19 to 1.76)		N/A		N/A		N/A	
	*P* value	<.001		.11		N/A		N/A		N/A	
	Cohen *d*	2.03		0.14		N/A		N/A		N/A	
**SAS^i^**											
	Baseline, mean (SD)	44.08 (10.44)		45.41 (7.71)		–1.33 (–3.61 to 0.95)		.25		–0.14	
	Postintervention, mean (SD)	30.37 (7.82)		44.53 (6.91)		–14.16 (–16.00 to –12.33)		<.001		–1.92	
	Difference (95% CI)	13.71 (12.11 to 15.30)		0.88 (–0.05 to 1.81)		N/A		N/A		N/A	
	*P* value	<.001		.06		N/A		N/A		N/A	
	Cohen *d*	1.51		0.40		N/A		N/A		N/A	
**AIS^j^**											
	Baseline, mean (SD)	8.98 (3.45)		8.67 (3.08)		0.31 (–0.50 to 1.12)		.45		0.09	
	Postintervention, mean (SD)	7.52 (2.99)		8.27 (3.22)		–0.75 (–1.52 to 0.03)		.05		–0.24	
	Difference (95% CI)	1.45 (1.04 to 1.86)		0.40 (–0.04 to 0.83)		N/A		N/A		N/A	
	*P* value	<.001		.07		N/A		N/A		N/A	
	Cohen *d*	0.63		0.16		N/A		N/A		N/A	

^a^cCBT: computerized cognitive behavioral therapy.

^b^TAU: treatment as usual.

^c^*P* value of independent-samples *t* test.

^d^HAMD_17_: Hamilton Depression Rating Scale.

^e^N/A: not applicable.

^f^*P* value of paired *t* test.

^g^HAMA: Hamilton Anxiety Scale.

^h^SDS: Self-Rating Depression Scale.

^i^SAS: Self-Rating Anxiety Scale.

^j^AIS: Athens Insomnia Scale.

Exploratory subgroup analyses examined the influence of gender, age, and education level on treatment response. For both male and female patients, the cCBT + TAU group showed some evidence for a greater reduction in HAMD_17_, HAMA, SDS, and SAS scores compared with the TAU group. For female patients, we found no evidence of significant between-group differences for the change of AIS scores (Table S1 in Multimedia Appendix 1). Additionally, the cCBT + TAU group showed some evidence of greater improvement in symptoms of anxiety and depression compared with the TAU group, regardless of education level. However, changes in sleeping, as measured using AIS, did not differ between the cCBT + TAU group and TAU group for patients with middle school education (Table S2 in Multimedia Appendix 2). Additionally, the cCBT + TAU group also showed significantly decreased scores on all 5 scales compared with the TAU group, regardless of age (Table S3 in Multimedia Appendix 3).

Details about sociodemographic characteristics of participants who completed the 1-month follow-up assessment for each group are presented in Table 1. The results indicated that there were no significant differences between the cCBT + TAU group and TAU group for any of these variables. Besides, compared to the full sample in each condition, the follow-up sample showed no significant differences in demographic and psychological characteristics (Table 3). The patients who finished the follow-up assessment were analyzed using the MMRM and Bonferroni post hoc multiple comparison, with HAMD_17_, HAMA, SDS, SAS, and AIS scores as the dependent variables, to reveal the relationship between the two groups and three periods.

**Table 3:** Comparison in demographic and psychological characteristics between full sample and follow-up sample.

Characteristics			cCBT^a^ + TAU^b^ group														TAU group												
			Full sample		Follow-up sample		Difference (95% CI)			*P* value			Cohen *d* or φ			Full sample			Follow-up sample			Difference (95% CI)			*P* value			Cohen *d* or φ	
**Sex, n**								N/A^c^			.52			0.05^d^									N/A			.95			<0.01^d^
	Male	70		31											80			31											
	Female	56		20											46			18											
Age (years), mean (SD)			43.76 (14.31)		42.26 (12.66)		1.50 (–2.98 to 5.97)			.51			0.11			41.52 (11.51)			42.10 (10.58)			–0.58 (–4.29 to 3.14)			.76			–0.05	
Education (years)			10.68 (3.91)		10.57 (4.21)		0.12 (–1.18 to 1.41)			.86			0.03			10.67 (4.39)			10.72 (4.79)			–0.05 (–1.54 to 1.43)			.94			–0.01	
HAMD_17_^e^			15.13 (3.33)		15.28 (2.23)		–0.15 (–1.16 to 0.81)			.73			–0.05			15.52 (3.43)			15.70 (1.28)			–0.18 (–1.17 to 0.80)			.71			–0.07	
HAMA^f^			14.52 (3.13)		14.26 (2.31)		0.26 (–0.75 to 1.12)			.70			0.09			13.97 (2.72)			13.88 (1.86)			0.09 (–0.74 to 0.92)			.83			0.04	
SDS^g^			45.89 (8.78)		46.10 (7.59)		–0.21 (–2.81 to 2.67)			.96			–0.03			45.66 (8.28)			45.22 (8.15)			0.44 (–2.28 to 3.19)			.75			0.05	
SAS^h^			44.08 (10.44)		44.30 (10.49)		–0.22 (–3.13 to 3.66)			.88			–0.02			45.41 (7.71)			45.56 (7.25)			–0.15 (–2.65 to 2.35)			.91			–0.02	
AIS^i^			8.98 (3.45)		8.58 (2.95)		0.41 (–0.82 to 1.34)			.64			0.12			8.67 (3.08)			8.20 (1.29)			0.47 (–0.42 to 1.36)			.30			0.20	

^a^cCBT: computerized cognitive behavioral therapy.

^b^TAU: treatment as usual.

^c^N/A: not applicable.

^d^Indicates φ values.

^e^HAMD_17_: Hamilton Depression Rating Scale.

^f^HAMA: Hamilton Anxiety Scale.

^g^SDS: Self-Rating Depression Scale.

^h^SAS: Self-Rating Anxiety Scale.

^i^AIS: Athens Insomnia Scale.

As demonstrated in Table 4, a significant difference in the interaction between groups and periods (all *P*<.001) was observed in all scale scores. Post hoc analysis revealed significant reduction in all five scores during the postintervention and follow-up period in the cCBT + TAU group from baseline (all *P*<.05, adjusted by the Bonferroni method).

**Table 4:** Repeated measures analysis of variance results for scores of HAMD_17_, HAMA, SDS, SAS, and AIS between cCBT + TAU group and TAU group at baseline, postintervention, and follow-up.

Measure		cCBT^a^ + TAU^b^ group (n=51)	TAU group (n=50)	Difference (95% CI)	*P* value^c^	Partial η^2^
**HAMD_17_^d^**						
	Baseline, mean (SD)	15.28 (2.23)	15.70 (1.28)	–0.42 (–1.22 to 0.38)	.30	0.02
	Postintervention, mean (SD)	7.86 (3.04)^e^	15.46 (1.76)	–7.60 (–8.61 to –6.60)	<.001	0.82
	Follow-up, mean (SD)	6.86 (1.77)^e,f^	15.26 (2.32)	–8.40 (–9.30 to –7.50)	<.001	0.87
	*P* value	<.001^g^	<.001^h^	<.001^i^	N/A^j^	N/A
**HAMA^k^**						
	Baseline, mean (SD)	14.26 (2.31)	13.88 (1.86)	0.38 (–0.41 to 1.17)	.34	0.02
	Postintervention, mean (SD)	7.38 (2.84)^e^	13.24 (2.26)	–5.86 (–6.83 to –4.89)	<.001	0.75
	Follow-up, mean (SD)	6.10 (2.04)^e,f^	13.20 (1.96)	–7.10 (–7.81 to –6.39)	<.001	0.89
	*P* value	<.001^g^	<.001^h^	<.001^i^	N/A	N/A
**SDS^l^**						
	Baseline, mean (SD)	46.10 (7.59)	45.22 (8.15)	0.88 (–2.48 to 4.24)	.60	0.01
	Postintervention, mean (SD)	32.56 (6.54)^e^	45.56 (6.59)	–13.00 (–15.80 to –10.20)	<.001	0.64
	Follow-up, mean (SD)	31.14 (5.65)^e,f^	44.70 (6.05)	–13.56 (–15.98 to –11.14)	<.001	0.72
	*P* value	<.001^g^	<.001^h^	<.001^i^	N/A	N/A
**SAS^m^**						
	Baseline, mean (SD)	44.30 (10.49)	45.56 (7.25)	–1.26 (–4.97 to 2.45)	.50	0.01
	Postintervention, mean (SD)	29.66 (7.31)^e^	45.52 (7.05)	–15.86 (–18.97 to –12.75)	<.001	0.68
	Follow-up, mean (SD)	29.12 (6.08)^e^	44.92 (5.92)	–15.80 (–18.34 to –13.27)	<.001	0.76
	*P* value	<.001^g^	<.001^h^	<.001^i^	N/A	N/A
**AIS^n^**						
	Baseline, mean (SD)	8.58 (2.95)	8.20 (1.29)	0.38 (–0.51 to 1.27)	.40	0.02
	Postintervention, mean (SD)	6.98 (2.99)^e^	8.00 (2.22)	–1.02 (–2.11 to 0.07)	.06	0.07
	Follow-up, mean (SD)	6.88 (2.72)^e^	7.82 (2.17)	–0.94 (–1.84 to –0.04)	.04	0.08
	*P* value	<.001^g^	<.001^h^	.002^i^	N/A	N/A

^a^cCBT: computerized cognitive behavioral therapy.

^b^TAU: treatment as usual.

^c^*P* value adjusted by the Bonferroni method of between-group differences.

^d^HAMD_17_: Hamilton Depression Rating Scale.

^e^Compared with baseline, *P*<.05, adjusted by the Bonferroni method.

^f^Compared with postintervention, *P*<.05, adjusted by the Bonferroni method.

^g^*P* value of group effect in the mixed-effects model for repeated measures.

^h^*P* value of time effect in the mixed-effects model for repeated measures.

^i^*P* value of interactive effects between time and group in the mixed-effects model for repeated measures.

^j^N/A: not applicable.

^k^HAMA: Hamilton Anxiety Scale.

^l^SDS: Self-Rating Depression Scale.

^m^SAS: Self-Rating Anxiety Scale.

^n^AIS: Athens Insomnia Scale.

## Discussion

### Principal Results

This study tested the effectiveness of the cCBT program we developed in patients with COVID-19 who have mild to moderate depressive or anxiety symptoms, compared with the TAU condition. The results implicated that the cCBT intervention was beneficial, given significant between-group differences in depression (HAMD_17_, SDS), anxiety (HAMA, SAS), and insomnia (AIS). Other notable between-group observations were no significant differences in the improvement of insomnia among females and those with middle school education.

Facing public health emergencies, people experiencing stress responses are likely to encounter mental health problems [39]. The pandemic has increased the risk of psychological disorders, especially in confirmed cases of COVID-19 [40]. A meta-analysis about the prevalence of mental health problems in patients with COVID-19 showed prevalence rates of depression at 15.97%, anxiety at 15.5%, PTSD at 21.94%, and insomnia at 23.87% [41]—all far higher than usual [42]. Another survey of early rehabilitation on patients with COVID-19 showed that 43.1% of them had flashback symptoms, 27.8% had voiding symptoms, 40.5% had increased alertness, 22.2% had anxiety, and 38.1% had depression [43]. It can be implied that the main mental health problems of patients with COVID-19 were depression, anxiety, and PTSD. Selective serotonin reuptake inhibitor antidepressants are the first-line drug treatment for depression, anxiety, and PTSD [44,45]. Nevertheless, in the conditions of the pandemic, medication for COVID-19–prompted trauma responses is not the best option because it takes effect slowly. Moreover, only an approximate 33% of the patients were clinically cured [46], and these demonstrated residual symptoms following acute treatment [47]. Further, a review indicated there is a great potential for drug-to-drug interactions between antiretroviral drugs and psychotropics, especially antidepressants and anxiolytics, which could hinder treatment efficacy or even produce life-threatening adverse drug reactions [48].

As such, nonpharmacological treatment is considered an effective and safe option without adverse effects. CBT is the first-line psychological treatment recommended by the guidelines of depression, anxiety, and PTSD (ie, Canadian Network for Mood and Anxiety Treatments and National Institute of Health and Care Excellence guideline [49,50]. Li et al [51] designed a randomized controlled trial to prove traditional CBT significantly decreased depression, anxiety, and stress symptoms in patients with COVID-19 [51]. However, administering CBT effectively using face-to-face techniques is very time-consuming and costly, and is unavailable to most patients in isolation [52].

In this study, the independently developed cCBT program for patients with COVID-19 was used as a systematic cognitive, mood, and behavior intervention. Through this program, selected patients were subjected to 7 interventions for a period of 1 week, and each intervention was divided into three steps. Step 1 was designed to help patients acquire knowledge of novel coronavirus pneumonia, recognize their own stress statuses, learn the methods of adjusting negative emotions, educate themselves about postisolation, and practice behavioral relaxation training. Step 2 was designed using several games to reinforce the knowledge acquired from Step 1. Step 3 was designed to guide the patients in relaxation techniques.

The cCBT program we developed was used in five hospitals in China that isolated patients with COVID-19 during the outbreak. A total of 252 patients with COVID-19 were collected in this study and randomly divided into a cCBT + TAU group (n=126) and a TAU group (n=126); 51 patients in the cCBT + TAU group and 50 patients in the TAU group finished the follow-up assessment in one of the centers. We observed that the symptoms of depression, anxiety, and insomnia were relieved significantly in the patients who underwent the cCBT program. The results were consistent with other short-term studies about online or digital CBT treatment of mental health problems among patients with COVID-19 in the pandemic [53,54]. In the 1-month follow-up, the results of our study still showed a significant improvement of the efficacy in the cCBT + TAU group, which was in accordance with previous research on cCBT relieving depression, anxiety, and insomnia symptoms in the short-term follow-up [55,56]. However, some studies revealed that the efficacy of cCBT was no different from the control group after a long-term follow-up [57,58]. Mental status was influenced by various factors such as environment, social and family support, and psychological adjustment. Therefore, long-term follow-up needs to be further explored.

We subsequently explored subgroup analyses examining the influence of gender, age, and education level on treatment response. Compared to the TAU group, insomnia symptoms were not improved significantly in female patients and those with middle school education treated by cCBT. Previous studies indicated that females were a high-risk population for insomnia [59]. A meta-analysis revealed the prevalence of insomnia in females was higher than that in males [60]; also, females were the predicted risk factor of insomnia during the COVID-19 pandemic [61,62]. Our results supported previous studies. Zhang et al [63] investigated the relationship between insomnia and education levels in Chinese females, and they found there was a negative correlation between them. Another research study obtained the opposite result, which reported that higher education levels were associated with sleep disorders [62]. Notably, an investigation that focused on patients with COVID-19 who were treated in the Fangcang shelter hospital in Wuhan, China found that lower education levels were more likely to prompt depression, anxiety, and stress but had no impact on insomnia [64]. There are great differences in the results of previous research studies; the correlation between education levels and insomnia remains unclear, and our study provided a little evidence of that.

### Advantages and Limitations

This study has several strengths. First, the cCBT program we independently developed is focused on patients with COVID-19 and is more suitable for the psychological adjustment of the patients than an ordinary cCBT program. Second, the use of a strict inclusion and exclusion standard ensured the homogeneity of participants. We controlled patient inclusivity through scores on HAMD_17_≥7 and <24 or HAMA≥7 and <21 to ensure that all patients had mild to moderate depressive and anxiety symptoms. The study involved five different hospitals from China to make the results representative. Finally, the patients were randomized to the cCBT + TAU group or the TAU group with a 1:1 ratio to balance confounding factors.

Inevitably, our trial has some limitations. First, participants in our trials were nonblinded, while the study looks at a multiplicity of outcomes, thereby increasing the risk for a type I error. We designed the trial initially to have patients and the evaluators blinded; however, to reduce the risk of infection, only a few doctors were permitted to enter the isolated ward. Such a situation impeded the use of blind methods in this study. Second, the sample sizes were relatively small, and the time before the follow-up was relatively short. In the future, we will continue to develop the cCBT program for a broader range of scenarios such as alleviating preoperative anxiety and depression in patients. Bigger sample sizes and a longer follow-up will be adopted to identify the long-term influence of cCBT, and the influencing factors of cCBT’s efficacy will be analyzed in more detail. Additionally, we did not account for more confounding factors such as drug side effects and related adversity, which may be also highly relevant to the symptoms of depression and anxiety.

### Conclusions

In sum, this was a prospective, multicenter, two-arm, parallel-group, and randomized controlled trial to identify the efficacy of a short-term cCBT program for treating symptoms of depression, anxiety, and insomnia among patients with COVID-19. The results suggested that the cCBT was significantly effective in relieving symptoms of depression, anxiety, and insomnia in patients with COVID-19 post intervention or after a 1-month follow-up. However, the insomnia symptoms in females and those with middle school education were more difficult to improve. Further research is needed to expand the sample size and to investigate the long-term effects of cCBT for symptoms of depression, anxiety, and insomnia.

## Appendix

Multimedia Appendix 1 Differences in the dependent variables after intervention between the treatment and control groups of males and females.

Multimedia Appendix 2 Differences in the dependent variables after intervention between the treatment and control groups of different education levels.

Multimedia Appendix 3 Differences in the dependent variables after intervention between the treatment and control groups of different ages.

Multimedia Appendix 4 CONSORT-EHEALTH checklist (V 1.6.1).

## Abbreviations

AIS: Athens Insomnia Scale
CBT: cognitive behavioral therapy
cCBT: computerized cognitive behavioral therapy
HAMA: Hamilton Anxiety Scale
HAMD_17_: Hamilton Depression Rating Scale
ICC: intraclass correlation coefficient
MERS: Middle East respiratory syndrome
MMRM: mixed-effects model for repeated measures
PTSD: posttraumatic stress disorder
SARS: severe acute respiratory syndrome
SAS: Self-Rating Anxiety Scale
SDS: Self-Rating Depression Scale
TAU: treatment as usual
