# Supplementary material for: The Efficacy of Computerized Cognitive Behavioral Therapy for Depressive and Anxiety Symptoms in Patients With COVID-19: Randomized Controlled Trial
Source: J Med Internet Res. 2021 May 14;23(5):e26883. doi: 10.2196/26883 (PMC8128049; doi:10.2196/26883)
Supplement: Multimedia Appendix 1 [file jmir_v23i5e26883_app1.doc]

Table S1. Differences in dependent variables after intervention between the treatment and control groups of male and female respectively

|  |  | cCBT+TAU group | TAU group | t | p | ES |
| --- | --- | --- | --- | --- | --- | --- |
| Male | N | 70 | 80 |  |  |  |
|  | HAMD_-17_ |  |  |  |  |  |
|  | Baseline | 15.16± 3.25 | 15.35± 3.34 | -0.36 | 0.721 |  |
|  | Post-intervention | 8.09± 3.46 | 14.74± 3.578 | -11.53 | <0.001 | 1.89 |
|  | Change | -7.07± 3.21 | -0.61± 1.84 | -14.86 | <0.001 | 2.47 |
|  | HAMA |  |  |  |  |  |
|  | Baseline | 14.50± 3.13 | 13.95± 2.32 | 1.21 | 0.230 |  |
|  | Post-intervention | 7.39± 3.16 | 13.55± 2.90 | -12.45 | <0.001 | 2.03 |
|  | Change | -7.11± 3.17 | -0.40± 2.32 | -14.64 | <0.001 | 2.42 |
|  | SDS |  |  |  |  |  |
|  | Baseline | 45.23± 9.35 | 46.40± 8.14 | -0.81 | 0.418 |  |
|  | Post-intervention | 31.40± 7.05 | 45.30± 7.26 | -11.86 | <0.001 | 1.94 |
|  | Change | -13.83± 6.62 | -1.10± 5.79 | -12.56 | <0.001 | 2.05 |
|  | SAS |  |  |  |  |  |
|  | Baseline | 44.36± 10.74 | 46.85± 7.34 | -1.64 | 0.105 |  |
|  | Post-intervention | 30.74± 8.24 | 45.73± 6.83 | -12.03 | <0.001 | 1.98 |
|  | Change | -13.61± 9.08 | -1.13± 5.72 | -9.92 | <0.001 | 1.65 |
|  | AIS |  |  |  |  |  |
|  | Baseline | 9.21± 3.36 | 8.50± 2.90 | 1.40 | 0.164 |  |
|  | Post-intervention | 7.49± 2.87 | 8.14± 3.19 | -1.31 | 0.193 |  |
|  | Change | -1.73± 2.29 | -0.36± 2.69 | -3.34 | 0.001 | 0.55 |
| Female | N | 56 | 46 |  |  |  |
|  | HAMD_-17_ |  |  |  |  |  |
|  | Baseline | 15.09± 3.45 | 15.80± 3.61 | -1.02 | 0.310 |  |
|  | Post-intervention | 8.32± 3.66 | 16.00± 3.66 | -10.55 | <0.001 | 2.10 |
|  | Change | -6.77± 3.71 | 0.20± 2.26 | -11.66 | <0.001 | 2.27 |
|  | HAMA |  |  |  |  |  |
|  | Baseline | 14.55± 3.10 | 14.00± 3.33 | 0.87 | 0.388 |  |
|  | Post-intervention | 8.30± 4.05 | 13.78± 3.79 | -7.00 | <0.001 | 1.40 |
|  | Change | -6.25± 3.69 | -0.22± 2.49 | -9.46 | <0.001 | 1.92 |
|  | SDS |  |  |  |  |  |
|  | Baseline | 46.71± 8.01 | 44.37± 8.44 | 1.44 | 0.154 |  |
|  | Post-intervention | 32.64± 6.66 | 44.13± 7.87 | -7.98 | <0.001 | 1.58 |
|  | Change | -14.07± 7.20 | -0.24± 5.01 | -11.40 | <0.001 | 2.23 |
|  | SAS |  |  |  |  |  |
|  | Baseline | 43.73± 10.14 | 42.91± 7.77 | 0.46 | 0.645 |  |
|  | Post-intervention | 29.82± 7.31 | 42.46± 6.63 | -9.05 | <0.001 | 1.81 |
|  | Change | -13.91± 8.99 | -0.46± 4.39 | -9.86 | <0.001 | 1.90 |
|  | AIS |  |  |  |  |  |
|  | Baseline | 8.68± 3.57 | 8.96± 3.37 | -0.40 | 0.689 |  |
|  | Post-intervention | 7.57± 3.16 | 8.50± 3.30 | -1.45 | 0.150 |  |
|  | Change | -1.11± 2.32 | -0.46± 2.07 | -1.48 | 0.142 |  |

Abbreviations: cCBT = computerized cognitive behavioral therapy. TAU = Treatment as usual. HAMD-17 = Hamilton Depression Scale. HAMA = Hamilton Anxiety Scale. SDS = Self-rating Depression Scale. SAS = Self-Rating Anxiety Scale. AIS = Athens Insomnia Scale. ES: effect size.
