# Supplementary material for: The Efficacy of Computerized Cognitive Behavioral Therapy for Depressive and Anxiety Symptoms in Patients With COVID-19: Randomized Controlled Trial
Source: J Med Internet Res. 2021 May 14;23(5):e26883. doi: 10.2196/26883 (PMC8128049; doi:10.2196/26883)
Supplement: Multimedia Appendix 2 [file jmir_v23i5e26883_app2.doc]

Table S2 Differences in dependent variables after intervention between the treatment and control groups of different education level

|  |  | cCBT+TAU group | TAU group | t | p | ES |
| --- | --- | --- | --- | --- | --- | --- |
| ≤ Primary school | N | 35 | 37 |  |  |  |
|  | HAMD_-17_ |  |  |  |  |  |
|  | Baseline | 14.94± 3.17 | 16.16± 2.60 | -1.79 | .078 |  |
|  | Post-intervention | 7.49± 2.94 | 15.81± 2.94 | -12.00 | <.001 | 2.83 |
|  | Change | -7.46± 3.26 | -0.35± 1.84 | -11.31 | <.001 | 2.69 |
|  | HAMA |  |  |  |  |  |
|  | Baseline | 14.37± 3.45 | 13.62± 2.44 | 1.07 | .289 |  |
|  | Post-intervention | 7.40± 3.27 | 13.14± 2.96 | -7.82 | <.001 | 1.84 |
|  | Change | -6.97± 2.66 | -0.49± 2.14 | -11.42 | <.001 | 2.68 |
|  | SDS |  |  |  |  |  |
|  | Baseline | 44.71± 8.62 | 47.59± 6.86 | -1.57 | 0.120 |  |
|  | Post-intervention | 31.11± 6.62 | 46.30± 6.11 | -10.12 | <.001 | 2.38 |
|  | Change | -13.60± 6.84 | -1.30± 6.23 | -7.99 | <.001 | 1.88 |
|  | SAS |  |  |  |  |  |
|  | Baseline | 44.23± 11.25 | 48.08± 7.46 | -1.70 | .094 |  |
|  | Post-intervention | 29.23± 7.36 | 46.14± 6.03 | -10.69 | <.001 | 2.51 |
|  | Change | -15.00± 10.80 | -1.95± 6.33 | -6.21 | <.001 | 1.47 |
|  | AIS |  |  |  |  |  |
|  | Baseline | 8.63± 3.39 | 7.95± 2.53 | 0.97 | .334 |  |
|  | Post-intervention | 7.09± 2.88 | 7.70± 2.84 | -0.92 | .363 |  |
|  | Change | -1.54± 2.27 | -0.24± 2.47 | -2.33 | .023 | 0.55 |
| Middle school | N | 56 | 48 |  |  |  |
|  | HAMD_-17_ |  |  |  |  |  |
|  | Baseline | 15.52± 3.49 | 14.73± 3.81 | 1.10 | .273 |  |
|  | Post-intervention | 8.50± 3.67 | 14.75± 3.97 | -8.34 | <.001 | 1.64 |
|  | Change | -7.02± 3.47 | 0.02± 2.25 | -12.44 | <.001 | 2.41 |
|  | HAMA |  |  |  |  |  |
|  | Baseline | 14.84± 3.03 | 14.17± 3.27 | 1.09 | .279 |  |
|  | Post-intervention | 8.21± 3.93 | 14.02± 3.82 | -7.61 | <.001 |  |
|  | Change | -6.63± 3.77 | -0.15± 2.54 | -10.40 | <.001 |  |
|  | SDS |  |  |  |  |  |
|  | Baseline | 45.30± 9.49 | 44.15± 8.87 | 0.64 | .524 |  |
|  | Post-intervention | 31.61± 7.21 | 44.13± 9.04 | -7.72 | <.001 | 1.53 |
|  | Change | -13.70± 7.42 | -0.02± 4.95 | -11.19 | <.001 | 2.17 |
|  | SAS |  |  |  |  |  |
|  | Baseline | 43.34± 10.48 | 43.21± 7.03 | 0.08 | .940 |  |
|  | Post-intervention | 30.89± 8.26 | 40.00± 6.29 | -8.47 | <.001 | 1.24 |
|  | Change | -12.45± 8.79 | -0.21± 4.89 | -8.93 | <.001 | 1.72 |
|  | AIS |  |  |  |  |  |
|  | Baseline | 9.46± 3.28 | 9.33± 3.44 | 0.20 | .843 |  |
|  | Post-intervention | 8.29± 2.70 | 8.52± 3.38 | -0.39 | .699 |  |
|  | Change | -1.18± 2.24 | -0.81± 2.93 | -0.71 | .482 |  |
| ≥ College | N | 35 | 41 |  |  |  |
|  | HAMD_-17_ |  |  |  |  |  |
|  | Baseline | 14.69± 3.23 | 15.85± 3.52 | -1.50 | .139 |  |
|  | Post-intervention | 8.40± 3.86 | 15.17± 3.82 | -7.67 | <.001 | 1.76 |
|  | Change | -6.29± 3.52 | 0.68± 1.90 | -8.43 | <.001 | 2.46 |
|  | HAMA |  |  |  |  |  |
|  | Baseline | 14.17± 2.91 | 14.05± 2.25 | 0.21 | .836 |  |
|  | Post-intervention | 7.51± 3.39 | 13.63± 2.72 | -8.73 | <.001 | 1.99 |
|  | Change | -6.66± 3.59 | -0.41± 2.41 | -8.75 | <.001 | 2.05 |
|  | SDS |  |  |  |  |  |
|  | Baseline | 48.00± 7.54 | 45.68± 8.55 | 1.24 | .218 |  |
|  | Post-intervention | 33.34± 6.57 | 44.46± 6.52 | -7.39 | <.001 | 1.70 |
|  | Change | -14.66± 6.03 | -1.22± 5.49 | -10.16 | <.001 | 2.33 |
|  | SAS |  |  |  |  |  |
|  | Baseline | 45.11± 9.73 | 45.59± 8.08 | -0.23 | .818 |  |
|  | Post-intervention | 30.69± 7.63 | 44.88± 8.05 | -7.84 | <.001 | 1.81 |
|  | Change | -14.43± 7.39 | -0.71± 4.56 | -9.54 | <.001 | 2.23 |
|  | AIS |  |  |  |  |  |
|  | Baseline | 8.54± 3.77 | 8.54± 2.98 | 0.01 | .994 |  |
|  | Post-intervention | 6.74± 3.32 | 8.49± 3.36 | -2.27 | .026 | 0.52 |
|  | Change | -1.80± 2.48 | -0.05± 1.73 | -3.51 | .001 | 0.82 |

Abbreviations: cCBT = computerized cognitive behavioral therapy. TAU = Treatment as usual. HAMD-17 = Hamilton Depression Scale. HAMA = Hamilton Anxiety Scale. SDS = Self-rating Depression Scale. SAS = Self-Rating Anxiety Scale. AIS = Athens Insomnia Scale. ES: effect size.
