# Supplementary material for: The Efficacy of Computerized Cognitive Behavioral Therapy for Depressive and Anxiety Symptoms in Patients With COVID-19: Randomized Controlled Trial
Source: J Med Internet Res. 2021 May 14;23(5):e26883. doi: 10.2196/26883 (PMC8128049; doi:10.2196/26883)
Supplement: Multimedia Appendix 3 [file jmir_v23i5e26883_app3.doc]

Table S3 Differences in dependent variables after intervention between the treatment and control groups of different age

|  |  | cCBT+TAU group  (Mean± SD) | TAU group  (Mean± SD) | t | *P* | ES |
| --- | --- | --- | --- | --- | --- | --- |
| ≤ 30 years | N | 30 | 24 |  |  |  |
|  | HAMD_17_ |  |  |  |  |  |
|  | Baseline | 15.37± 3.37 | 15.71± 4.17 | -0.33 | .74 |  |
|  | Post-intervention | 9.47± 4.24 | 15.46± 4.76 | -4.88 | <.001 | 0.53 |
|  | Change | -5.90± 3.96 | -0.25± 1.60 | -6.57 | <.001 | 1.87 |
|  | HAMA |  |  |  |  |  |
|  | Baseline | 14.50± 3.07 | 13.92± 3.36 | 0.67 | .51 |  |
|  | Post-intervention | 7.20± 3.77 | 12.75± 3.93 | -5.28 | <.001 | 1.44 |
|  | Change | -7.30± 3.91 | -1.17± 3.16 | -6.22 | <.001 | 1.72 |
|  | SDS |  |  |  |  |  |
|  | Baseline | 46.03± 9.60 | 44.25± 7.77 | 0.74 | .46 |  |
|  | Post-intervention | 31.13± 7.31 | 44.71± 7.04 | -6.89 | <.001 | 1.89 |
|  | Change | -14.90± 8.20 | 0.46± 5.82 | -7.75 | <.001 | 2.16 |
|  | SAS |  |  |  |  |  |
|  | Baseline | 41.10± 9.94 | 46.17± 8.71 | -1.97 | .06 |  |
|  | Post-intervention | 30.77± 8.90 | 45.88± 9.00 | -6.17 | <.001 | 1.68 |
|  | Change | -10.33± 7.14 | -0.29± 5.66 | -5.62 | <.001 | 1.56 |
|  | AIS |  |  |  |  |  |
|  | Baseline | 9.40± 3.40 | 8.54± 2.73 | 1.00 | .32 |  |
|  | Post-intervention | 7.80± 3.32 | 8.25± 3.39 | -0.49 | .62 |  |
|  | Change | -1.60± 2.44 | -0.29± 1.87 | -2.16 | .03 | 0.60 |
| 30-50 years | N | 58 | 62 |  |  |  |
|  | HAMD_17_ |  |  |  |  |  |
|  | Baseline | 15.26± 2.86 | 15.44± 3.42 | -0.31 | .76 |  |
|  | Post-intervention | 7.59± 2.77 | 14.90± 3.33 | -13.05 | <.001 | 2.39 |
|  | Change | -7.67± 2.98 | -0.53± 1.76 | -16.09 | <.001 | 2.41 |
|  | HAMA |  |  |  |  |  |
|  | Baseline | 14.67± 2.76 | 14.06± 2.36 | 1.30 | .20 |  |
|  | Post-intervention | 8.47± 3.79 | 13.69± 2.56 | -8.92 | <.001 | 1.61 |
|  | Change | -6.21± 3.36 | -0.37± 2.01 | -11.65 | <.001 | 2.11 |
|  | SDS |  |  |  |  |  |
|  | Baseline | 47.43± 8.05 | 46.52± 8.58 | 0.60 | .55 |  |
|  | Post-intervention | 32.74± 7.01 | 44.69± 7.63 | -8.92 | <.001 | 1.63 |
|  | Change | -14.69± 6.08 | -1.82± 5.11 | -12.58 | <.001 | 2.29 |
|  | SAS |  |  |  |  |  |
|  | Baseline | 46.26± 10.10 | 46.15± 7.75 | 0.07 | .95 |  |
|  | Post-intervention | 29.31± 7.35 | 45.10± 6.71 | -12.30 | <.001 | 2.24 |
|  | Change | -16.95± 8.98 | -1.05± 5.30 | -11.90 | <.001 | 2.16 |
|  | AIS |  |  |  |  |  |
|  | Baseline | 8.31± 3.47 | 8.76± 2.87 | -0.77 | .55 |  |
|  | Post-intervention | 6.67± 2.89 | 8.27± 3.18 | -2.88 | .01 | 0.53 |
|  | Change | -1.64± 2.53 | -0.48± 2.80 | -2.36 | .02 | 0.43 |
| ≥ 50 years | N | 38 | 40 |  |  |  |
|  | HAMD_17_ |  |  |  |  |  |
|  | Baseline | 14.74± 3.96 | 15.53± 3.02 | -0.99 | .32 |  |
|  | Post-intervention | 8.11± 3.81 | 15.50± 3.40 | -9.05 | <.001 | 2.05 |
|  | Change | -6.63± 3.45 | -0.03± 2.59 | -9.60 | <.001 | 2.16 |
|  | HAMA |  |  |  |  |  |
|  | Baseline | 14.32± 3.70 | 13.85± 2.89 | -0.63 | .53 |  |
|  | Post-intervention | 7.24± 3.04 | 14.08± 3.69 | -8.90 | <.001 | 2.02 |
|  | Change | -7.08± 3.06 | 0.23± 2.26 | -12.03 | <.001 | 2.72 |
|  | SDS |  |  |  |  |  |
|  | Baseline | 43.42± 8.85 | 45.18± 8.14 | -0.91 | . 37 |  |
|  | Post-intervention | 31.39± 6.36 | 45.25± 7.67 | -8.66 | <.001 | 1.97 |
|  | Change | -12.03± 6.63 | 0.08± 5.76 | -8.62 | <.001 | 1.95 |
|  | SAS |  |  |  |  |  |
|  | Baseline | 43.11± 10.88 | 43.83± 6.95 | -0.35 | .73 |  |
|  | Post-intervention | 31.68± 7.58 | 42.85± 5.53 | -7.46 | <.001 | 1.68 |
|  | Change | -11.42± 9.02 | -0.98± 5.06 | -6.35 | <.001 | 1.43 |
|  | AIS |  |  |  |  |  |
|  | Baseline | 9.66± 3.36 | 8.60± 3.61 | 1.34 | .19 |  |
|  | Post-intervention | 7.89± 2.72 | 8.28± 3.27 | -0.56 | .58 |  |
|  | Change | -1.76± 2.45 | -0.33± 2.24 | -2.71 | .01 | 0.61 |

Abbreviations: cCBT = computerized cognitive behavioral therapy. TAU = Treatment as usual. HAMD_17_ = Hamilton Depression Scale. HAMA = Hamilton Anxiety Scale. SDS = Self-rating Depression Scale. SAS = Self-Rating Anxiety Scale. AIS = Athens Insomnia Scale. ES: effect size (Cohen’s d).
